# Greater adherence to the Planetary Health Diet is inversely associated with dyslipidemia in children: PASE Study—Brazil

**DOI:** 10.3389/fnut.2026.1684547

**Published:** 2026-03-12

**Authors:** Érica Priulli, Mariana de Santis Filgueiras, Dayane de Castro Morais, Bruna Clemente Cota, Juliana Farias de Novaes

**Affiliations:** Department of Nutrition and Health, Universidade Federal de Viçosa, Viçosa, Minas Gerais, Brazil

**Keywords:** planetary diet, eat-lancet, sustainable diet, children, dyslipidemia

## Abstract

**Background/objectives:**

Healthy and sustainable diets, such as the Planetary Health Diet (PHD), may contribute to the prevention and control of cardiovascular disease (CVD). However, there is a lack of evidence on this relationship in childhood. This study assessed the association between adherence to the PHD and lipid markers in children.

**Methods:**

Cross-sectional study with a representative sample of 378 children aged 8 and 9 in Viçosa, Minas Gerais, Brazil. The food consumption was assessed using three 24-h dietary recalls and the adherence to the PHD was measured through the Planetary Health Diet Index (PHDI). Dyslipidemia was defined by the presence of one or more altered lipid markers (total cholesterol—TC, triglycerides—TG, low-density lipoprotein—LDL-c, and high-density lipoprotein—HDL-c). Associations between PHDI and lipid outcomes were assessed using adjusted linear regression models.

**Results:**

The mean PHDI was 37.5 (SD: 10.6) and 70% of the children had dyslipidemia. The increase of one standard deviation (SD) of PHDI was associated with a reduction in serum concentrations of TC (*β* = −2.81, 95% CI: −5.57; −0.05) and LDL-c (*β* = −2.81, 95% CI: −5.07; −0.56). The Moderation component of the PHDI was also inversely associated with LDL-c (*β* = −2.88, 95% CI: −5.13; −0.64). In addition, the PHDI and its Adequacy and Ratio components showed inverse associations with the number of altered lipid markers (p-trend < 0.05). The PHDI was positively associated with carbohydrate and fiber intake, and inversely associated with calorie intake, saturated fat and dietary cholesterol.

**Conclusion:**

The greater adherence to the PHD was inversely associated with dyslipidemia in children, such as reduced TC, LDL-c, and fewer altered lipid markers, suggesting a potential benefit of adopting healthy and sustainable diet since childhood.

## 1 Introduction

Dyslipidemia in childhood, characterized by elevated concentrations of total cholesterol (TC), triglycerides (TG), low-density lipoprotein (LDL-c) and/or reduced concentrations of high-density lipoprotein (HDL-c) (1), is associated with the early development of atherosclerotic lesions (2), persisting into adulthood (3). In Brazil, the prevalence of dyslipidemia is high, affecting around 70% of children (4) and 78.2% of adolescents (5).

Historically, the dietary management of dyslipidemia emphasized specific nutrients, such as saturated fat and fiber, as well as isolated foods. In recent years, sustainable dietary patterns have been recognized as effective strategies in the prevention and treatment of dyslipidemia and cardiovascular disease (CVD) (6). Within this framework, a sustainable diet pattern offers additional benefits, contributing to the reduction of malnutrition, chronic diseases and the impacts of climate change on human health (7).

In 2019, the co-occurrence and interaction of obesity (including other chronic non-communicable diseases), undernutrition and climate change were described as Global Syndemic, highlighting the required integrated actions to address the common drivers of these three public health challenges (8). Therefore, the EAT-Lancet commission report proposed the Planetary Health Diet (PHD), a dietary pattern that prioritizes plant-based and whole foods, and limits the consumption of animal-based foods and added sugar (7).

Although evidence in adolescents and adults indicates that greater adherence to PHD is associated with lower TC concentrations (9, 10) and reduced risk of CVD mortality (11), there is a lack of studies evaluating this relationship in childhood. In this context, this study aimed to evaluate the association between adherence to PHD and lipid markers in children. The hypothesis is that greater adherence to PHD is inversely associated with dyslipidemia in childhood.

## 2 Materials and methods

### 2.1 Study design and participants

This is a cross-sectional study of the “Schoolchildren Health Assessment Survey” (PASE, in Portuguese) that aimed to assess the cardiometabolic risk and its associated factors in children from Viçosa, Minas Gerais, Brazil. In 2015, a random sample of 378 children aged 8 and 9 years old was selected, representative according to age and sex, from a total of 1,464 children in this age group enrolled in all public (*n* = 17) and private (*n* = 7) urban schools in the municipality. Viçosa is a city of approximately 72,000 inhabitants, located 227 km from Belo Horizonte, capital of Minas Gerais. Children with health conditions that affected nutritional status or body composition, those chronically using medications that interfered with glucose and/or lipid metabolism, and cases without successful contact with guardians after three attempts were not included in the study.

This study was conducted according to the guidelines established in the Declaration of Helsinki and approved by the Human Research Ethics Committee of the Universidade Federal de Viçosa (n° 663.171/2014). All guardians signed the Informed Consent Form.

### 2.2 Data collection

The information of sex, age, skin color, screen time (hours/day), per capita household income and family history of dyslipidemia was collected through a semi-structured questionnaire applied to parents or guardians. The skin color of participants was self-reported and categorized as White, Brown, Black, or Asian, according to Brazilian Institute of Geography and Statistics (IBGE).

The weight and height were measured to calculate the body mass index for age (BMI-for-age), classified in *z*-scores according to World Health Organization criteria (12). The body fat percentage was estimated by Dual-Energy X-Ray Absorptiometry (DXA) (Lunar Prodigy, GE Healthcare^®^, Madison, USA).

### 2.3 Food consumption

The food consumption was assessed using the average of three 24-h dietary recalls (R24h), applied two on weekdays and one at the weekend, with a minimal interval of 15 days between them. The interviews were conducted with the children, in the presence of their guardians, with the help of household utensils and a photo album (13) to facilitate the accuracy of food portion estimates. The Brazilian Food Composition Table, version 7.0 (14), was used to quantify nutrients. The TBCA was developed in accordance with the guidelines of the International Network of Food Data Systems (INFOODS). For recipe breakdown, we used a database containing nutritional composition and standardized Brazilian recipes, according to the TBCA (14).

The food consumption data were entered into the Diet Pro^®^ 5i software, version 5.8 (DIET PRO, 1997) and later exported to a Microsoft Excel^®^ spreadsheet (Microsoft Corp., Redmond, USA). The link to the Brazilian Food Composition Table (TBCA, in Portuguese) version 7.0 (14) was performed using the extensions of Power Query (15) and Visual Basic for Applications (16). All nutrients were presented by total caloric intake using the energy density method, expressed in grams or milligrams per 1,000 Kcal.

### 2.4 Planetary Health Diet Index

The Planetary Health Diet Index (PHDI) is a validated Brazilian instrument to assess adherence to the Planetary Health Diet (PHD) recommendations (17). First, all mixed dishes and processed foods were disaggregated into their basic ingredients using a Brazilian database of standard recipes. For highly processed products that are mainly composed of a single base ingredient, such as those made primarily from maize starch or wheat flour, the energy contribution of these ingredients was estimated based on their fat and added sugar content. This procedure was applied to most processed products, except for processed meats, which were categorized based on their main ingredient or commonly marketed formulation into the respective groups of red meat (e.g., sausage, ham, and salami) or chicken and substitutes (e.g., nuggets) (17).

The PHDI scores were calculated following the method described by Cacau et al. (17). The PHDI uses cutoff points based on energy intake to calculate the score for 16 food groups, organized into four components: 1) Adequacy (nuts and peanuts, legumes, fruits, vegetables and whole cereals), 2) Moderation (red meat, chicken and substitutes, animal fats and added sugars), 3) Ratio (ratio of the dark green vegetables, and red and orange vegetables to total vegetables), and 4) Optimum (eggs, dairy, fish and seafood, tubers and potatoes, and vegetable oils). After classifying the foods, the percentage of caloric contribution for each group was calculated (group calories ÷ total calories consumed on the day × 100). The Adequacy, Moderation and Optimum components can each reach up to 10 points, while the Ratio component reaches a maximum of 5 points, resulting in a total score from 0 to 150, with higher values indicating greater adherence. The details of the cutoff points and scoring criteria were previously described by the authors who developed and validated the PHDI in Brazil (17).

### 2.5 Lipid markers

The dyslipidemia was defined by the presence of one or more of the following altered parameters, according to Faludi (1): TC ≥ 170 mg/dL, LDL-c ≥ 110 mg/dL, TG ≥ 75 mg/dL and HDL-c < 45 mg/dL. The sum of the altered markers was classified on a scale from zero (no alterations) to four (presence of all alterations).

Blood samples were collected after 12 h of fasting at the Clinical Analysis Laboratory of the Health Division of the Universidade Federal de Viçosa and stored at −80 °C until analysis. The lipid markers were measured in serum using an automatic analyzer (BioSystems 200 Mindray^®^, Nanchang, China) and Bioclin^®^ reagent (Belo Horizonte, Brazil), following the manufacturer’s recommendations.

### 2.6 Data analysis

*Exposure*: Total score of the Planetary Health Diet Index (PHDI) and its components (Adequacy, Moderation, Ratio and Optimum).
*Outcome*: Presence and number of altered lipid markers (dyslipidemia).
*Covariates*: Age, sex, per capita household income, skin color, body fat percentage, total caloric intake, screen time, and family history of dyslipidemia.

The PHDI score and its components were analyzed as continuous variables and presented as mean and standard deviation (SD). In the descriptive analysis, the means (SD) or absolute and relative frequencies (n, %) were calculated for each variable, according to the PHDI terciles. Pearson’s chi-square test and linear regression were used to analyze proportions or means, respectively.

Student’s *t*-test was used to compare the means of PHDI according to lipid markers, classified as normal or high/low. Linear regression models were used to estimate associations between the PHDI (and its components) and lipid markers, as well as with energy and nutrient intake. The adjustment variables were selected based on the literature (4, 17, 18). Statistical analyses were carried out using Stata^®^ 17.0 software. Figures were created using R software version 4.3.2 (R Core Team, 2024) and ggplot2 package. The significance level considered was 5%.

## 3 Results

This study included 378 children with a mean age of 8.5 years [Standard Deviation (SD) = 0.5] and a mean per capita income of US$ 234.28 (SD = 267.48). The mean of PHDI score was 37.45 (SD: 10.58) points. An inverse association was observed between PHDI and LDL-c concentrations (p-trend = 0.039) (Table 1).

**Table 1:** Sociodemographic, lifestyle, anthropometric, body composition and lipid profile characteristics of children according to planetary health diet index (PHDI) (Viçosa, Minas Gerais, Brazil, 2015–2016).

Variable	PHDI terciles			
	1st (*n* = 126)	2nd (*n* = 126)	3rd (*n* = 126)	*p*-value
PHDI total score	26.00 (5.57)	37.70 (2.53)	48.64 (6.45)	
Sex^¶^				
Male	71 (39.23%)	54 (29.83%)	56 (30.94%)	0.064
Female	55 (27.92%)	72 (36.55%)	70 (35.53%)	
Skin color^¶^				
White	42 (35.29%)	42 (35.29%)	35 (29.42%)	0.838
Brown	72 (34.12%)	67 (31.76%)	72 (34.12%)	
Black	11 (25.58%)	15 (34.88%)	17 (39.54%)	
Asian	1 (20.00%)	2 (40.00%)	3 (40.00%)	
Screen time (hours/day)^¶^				
≤2	59 (29.80%)	71 (35.86%)	68 (34.34%)	0.289
>2	67 (37.22%)	55 (30.56)	58 (32.22%)	
Height-for-age (*z*)^‡^	0.48 (1.04)	0.64 (1.07)	0.51 (0.99)	0.619
BMI-for-age (*z*)^‡^	0.28 (1.36)	0.48 (1.46)	0.46 (1.40)	0.534
% BF^‡^	22.42 (9.68)	25.63 (10.64)	24.57 (9.71)	0.196
TC (mg/dL)^‡^	152.24 (28.67)	156.18 (26.36)	148.50 (23.57)	0.055
LDL-c (mg/dL)^‡^	86.67 (23.57)	90.20 (23.55)	84.03 (22.80)	**0.039**
HDL-c (mg/dL)^‡^	50.62 (10.58)	50.53 (10.09)	49.07 (9.27)	0.368
TG (mg/dL)^‡^	77.08 (31.09)	81.90 (39.26)	77.52 (35.53)	0.721
Family history of dyslipidemia^¶^				
No	96 (34.66%)	89 (32.13%)	92 (33.21%)	0.796
Yes	30 (29.70%)	37 (36.64%)	34 (33.66%)	

TC, total cholesterol; LDL-c, low-density lipoprotein; HDL-c, high-density lipoprotein; TG, triglycerides; % BF, body fat; BMI, body mass index.

^δ^Approximate exchange rate of the real (R$) to the dollar (USD) at the time of this study (R$1.00 = USD 3.33).

^¶^*n* (%); Pearson’s *χ*^2^.

Mean (standard deviation). ^‡^Linear regression models with PHDI terciles as continuous predictors and variables of anthropometric, body composition, or lipid profile as continuous outcomes (*p*-trend).

Bold values indicate statistical significance (*p* < 0.05).

**Figure 1:**
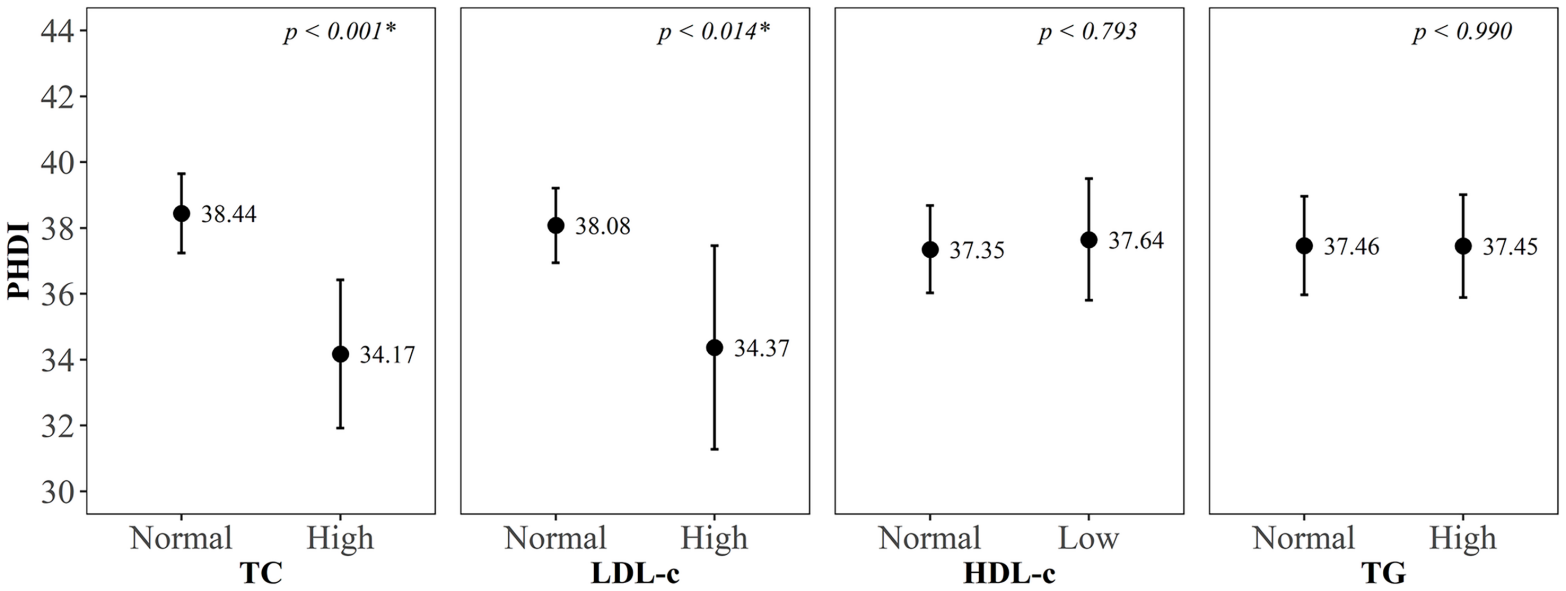
Means and standard deviations of the Planetary Health Diet Index (PHDI) score according to markers of dyslipidemia in children (Viçosa, Minas Gerais, Brazil, 2015–2016). TC, total cholesterol; LDL-c, low-density lipoprotein; HDL-c, high-density lipoprotein; TG, triglycerides. Student’s *t*-test (*p* < 0.05*).

We observed that 70% of the children (*n* = 263) presented dyslipidemia. The prevalence of high TC, LDL-c and TG, and low HDL-c, were, respectively, 23.1, 15.4, 47.2, and 35.0% (Supplementary Figure S1). The mean PHDI scores were lower in children with elevated TC and LDL-c, when compared to those with normal values of these markers (Figure 1).

**Figure 2:**
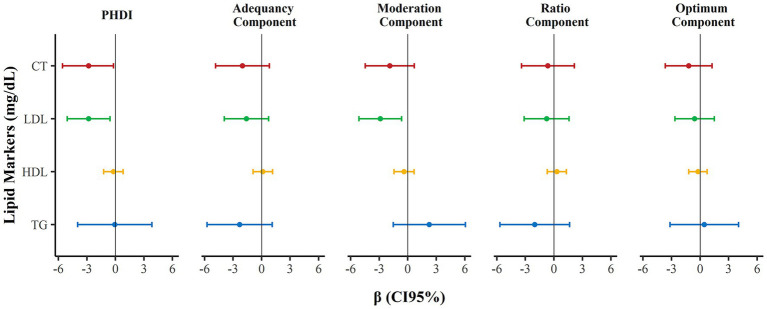
Association of the Planetary Health Diet Index (PHDI) and its components (adequacy, moderation, ratio and optimum) with markers of dyslipidemia in children (Viçosa, Minas Gerais, Brazil, 2015–2016). Linear regression with PHDI and its components as exposure and markers of dyslipidemia as outcome. Adjustment for age, sex, per capita household income, skin color, body fat percentage, total caloric intake, screen time, and family history of dyslipidemia. Robust variance estimates were specified in all models (*p* < 0.05).

For each increase of one standard deviation in the PHDI, reductions were identified in the concentrations of TC (*β* = −2.81, 95% CI: −5.57; −0.05, p-trend = 0.046) and LDL-c (*β* = −2.81, 95% CI: −5.07; −0.56, p-trend = 0.015). Additionally, each increase of one standard deviation in the Moderation component of the PHDI was associated with a reduction in LDL-c (*β* = −2.88, 95% CI: −5.13; −0.64, p-trend = 0.012). These associations were not found for HDL-c and TG (Figure 2).

The total PHDI score (*β* = −0.15, 95% CI: −0.26; −0.04), the Adequacy component (*β* = −0.16, 95% CI: −0.27; −0.06), and the Ratio component (*β* = −0.15, 95% CI: −0.25; −0.04) were inversely associated with the highest number of dyslipidemia markers (Table 2).

**Table 2:** Association of the planetary health diet index (PHDI) and its components with the number of markers of dyslipidemia (total cholesterol, low-density lipoprotein, high-density lipoprotein and/or triglycerides) in children (Viçosa, Minas Gerais, Brazil, 2015–2016).

PHDI and its components	Maximum score	Number of dyslipidemia markers						
		0 30.1% (*n* = 113)	1 33% (*n* = 124)	2 26.1% (*n* = 98)	3 7.7% (*n* = 29)	4 3.2% (*n* = 12)	Per 1 SD^‡^	p-trend
PHDI total score	150	38.86 (10.46)	37.18 (10.35)	37.96 (10.16)	33.90 (11.60)	34.31 (12.73)	**−0.15 (−0.26; −0.04)**	**0.007**
Adequacy component	50	16.95 (4.79)	15.72 (4.81)	16.06 (5.10)	14.55 (5.40)	14.74 (5.92)	**−0.16 (−0.27; −0.06)**	**0.003**
Ratio component	10	4.38 (2.35)	4.08 (2.20)	4.11 (2.37)	3.80 (2.46)	2.70 (2.70)	**−0.15 (−0.25; −0.04)**	**0.007**
Moderation component	40	5.23 (5.54)	5.85 (5.79)	5.57 (5.63)	4.64 (5.47)	5.07 (5.61)	−0.04 (−0.13; 0.06)	0.484
Optimum component	50	12.29 (6.90)	11.54 (5.80)	12.22 (6.07)	10.40 (5.82)	11.80 (6.78)	−0.03 (−0.14; 0.07)	0.542

SD (Standard deviation) ^**‡**^Linear regression with PHDI and its components (per 1 SD) as continuous predictors and the number of dyslipidemia markers as a continuous outcome. Robust variance estimates were specified in all models (*p* < 0.05).

Adjustment for age, sex, per capita household income, skin color, body fat percentage, total caloric intake, screen time, and family history of dyslipidemia.

Bold values indicate statistical significance (*p* < 0.05).

In addition, a higher PHDI score was associated with lower consumption of total calories (*β* = −47.76; 95% CI: −86.20; −9.32), saturated fat (*β* = −1.37; 95% CI: −1.95; −0.80) and cholesterol (*β* = −24.58; 95% CI: −36.02; −13.14). On the other hand, positive associations were observed between PHDI and consumption of carbohydrates (*β* = 3.11; 95% CI: 1.09, 5.13) and fibers (*β* = 1.67; 95% CI: 1.25, 2.09) (Table 3).

**Table 3:** Association between the planetary health diet index (PHDI) and daily nutrient intake in children (Viçosa, Minas Gerais, Brazil, 2015–2016).

Nutrients*	PHDI terciles (mean and standard deviation)			Per 1 SD	p-trend
	1st (26.00 ± 5.57) (*n* = 126)	2nd (37.70 ± 2.53) (*n* = 126)	3rd (48.64 ± 6.45) (*n* = 126)		
Energy (kcal)	1644.42 (401.66)	1628.44 (447.46)	1542.35 (385.99)	**−47.76 (−86.20; −9.32)**	**0.015**
Carbohydrate (g)	141.32 (18.07)	144.37 (16.67)	148.49 (16.62)	**3.11 (1.09; 5.13)**	**0.003**
Fiber (g)	8.93 (2.94)	10.44 (3.04)	12.11 (4.21)	**1.67 (1.25; 2.09)**	**<0.001**
Protein (g)	36.84 (10.65)	35.63 (8.66)	35.85 (7.33)	−0.03 (−1.07; 1.01)	0.955
Total fat (g)	44.27 (25.49)	39.53 (13.47)	35.88 (14.96)	−2.34 (−5.11; 0.42)	0.096
SFA (g)	15.35 (5.24)	13.24 (3.32)	11.95 (3.55)	**−1.37 (−1.95; −0.80)**	**<0.001**
MUFA (g)	13.08 (6.80)	11.90 (3.85)	10.73 (4.12)	−0.63 (−1.37; 0.11)	0.097
PUFA (g)	11.18 (13.39)	10.37 (7.03)	9.54 (7.86)	0.03 (−1.41; 1.47)	0.965
Trans fat (g)	1.17 (0.67)	0.98 (0.66)	0.99 (0.62)	−0.05 (−0.11; 0.01)	0.134
Cholesterol (mg)	195.13 (108.38)	154.54 (70.14)	130.79 (67.56)	**−24.58 (−36.02; −13.14)**	**<0.001**

SFA, saturated fatty acid; MUFA, monounsaturated fatty acid; PUF, polyunsaturated fatty acid; SD, standard deviation; T, tercile.

^*^Nutrients in grams or milligrams per 1,000 kcal.

Linear regression with ordinal categories of PHDI introduced into the model as a continuous predictor (per 1 SD), and energy and nutrients as outcomes.

Adjusted for age and sex.

Robust variance estimation specified in the models (*p* < 0.05).Bold values indicate statistically significant results (*p* < 0.05).

## 4 Discussion

This study confirms the hypothesis that children with higher adherence to PHDI presented lower serum concentrations of TC and LDL-c, and low number of altered lipid markers. These results reinforce that a more sustainable diet can also benefit cardiovascular health in childhood.

Our findings are consistent with the multicenter study “Healthy Lifestyle in Europe by Nutrition in Adolescents” (HELENA), which demonstrated an association between higher PHDI scores and lower probability of hypercholesterolemia in European adolescents (10). Similarly to our study, in another investigation developed with Brazilian adults (9), no significant associations were found of PHDI with serum TG and HDL-c concentrations. Contrasting with our findings, in the “National Health and Nutrition Examination Survey” (NHANES 2015–2018) with adults from the United States (≥20 years), the adherence to the PHD was associated with lower TG and higher HDL-c levels (19). Therefore, although the PHD contributes to a lower atherogenic risk, its influence on the lipid profile may differ according to age and geographic region.

Moreover, we found high prevalence of dyslipidemia in our sample (70%) and this finding is consistent with other study involving Brazilian adolescents (78.2%) (5) and children aged 2 to 9 years old (68,4%) (20), although different cutoff points were used to classify the lipid markers (1). Diet and physical inactivity are the main causes of dyslipidemia in childhood, except for cases of genetic dyslipidemia, which require a specific approach (1). In our sample, we identified that 27% of the participants had a family history of dyslipidemia and 48% presented sedentary behavior, demonstrated by screen time exceeding 2 h per day. Furthermore, the children presented high consumption of ultra-processed foods (21) and low PHDI score.

We observed that the total PHDI score, as well as its Adequacy and Ratio components, were associated with lower number of altered lipid markers, while the Moderation component showed an inverse association with LDL-c. The Adequacy component reflects greater consumption of foods that promote health and sustainability, such as fruits, vegetables, whole grains, legumes and nuts and peanuts. The Ratio component assesses the proportion of dark green vegetables and red-orange vegetables in relation to the total vegetables consumed, an indicator linked to the diversity and quality of the diet (17). Furthermore, the Moderation component, which presents an inverse score, reflects a lower quality and sustainability of diets, being composed of red meat, chicken and substitutes, animal fats and added sugars (17).

In this context, the benefits of higher PHDI scores and the Adequacy and Ratio components can be attributed to the higher intake of fiber, micronutrients, bioactive compounds and unsaturated fatty acids present in the predominant foods in these groups (6), in addition to the lower intake of animal fats and sugars, present in the foods in the Moderation group (17). In other previous study, our research group found that the inflammatory diet (rich in ultra-processed foods) was associated with atherogenic profile in children, confirming that the unhealthy diet can contribute to the worsening of lipid markers (4, 21).

The benefits of a sustainable diet for improved TC and LDL-c parameters may be explained by the inverse association between PHDI and intake of saturated fatty acids and cholesterol, as well as the positive association between PHDI and fiber intake, as demonstrated in this study. These findings are consistent with other study conducted with Brazilian adults, in which greater adherence to the PHDI was associated with similar nutritional profiles (17).

Furthermore, observational studies have indicated that the PHD, characterized by a high intake of fiber, unsaturated fats, fruits, and vegetables, was associated with lower risk of mortality from cardiovascular diseases (11), and better clinical conditions, including lower BMI, waist circumference (22), blood pressure, TC (9, 10), LDL-c, non–HDL-cholesterol (9), and triglycerides, as well as higher HDL-c levels (19). In contrast, a diet characterized by the Western dietary pattern, rich in highly processed, refined foods, red and processed meats, added sugars and saturated and trans fats, in addition to low consumption of fruits, vegetables, whole grains and nuts, has been associated with higher serum concentrations of LDL-c and inflammatory markers, and lower HDL-c, contributing to the development of the atherosclerotic process (23).

The greater adherence to the Mediterranean diet has beneficial effects on TC, LDL-C, HDL-C, and TG in childhood, as demonstrated by meta-analysis of intervention studies (24); in addition, the PHDI was positively associated with this dietary pattern (25). These results underscore the importance of adhering to a healthy diet, which, although not widely followed in many regions (26, 27), is crucial for cardiovascular health. Additionally, the PHDI was positively associated with the Diet Quality Index – Revised for the Brazilian population (IQD-R) and inversely associated with estimated dietary greenhouse gas emissions (17) and ultra-processed food consumption (28). These findings emphasize the relevance of considering sustainability in dietary guidelines, promoting benefits for human and planetary health.

This study has some limitations. The PHDI was developed and validated for Brazilian adults, and its application to children requires specific validation. However, its formula based on percentages of total caloric value allows it to be adapted to different age groups. Thus, considering the recent approach of the PHD and the scarcity of studies in children, this investigation contributes to demonstrating the possible benefits of the PHD in the prevention of childhood dyslipidemia, reinforcing the need for new validation studies of this index for the pediatric public. In addition, the cross-sectional design prevents the inference of causal relationships between the variables.

Positive aspects of this study include the use of three 24-h recalls, supported by a photo album to better estimate portion sizes, and the use of a Brazilian database to standardize the breakdown of recipes and classification of the PHDI food groups (14). In addition, the statistical analyses were adjusted for potential confounding factors.

Finally, although the associations between PHDI and markers of dyslipidemia appear small, they may have clinical relevance. A meta-analysis suggested that a reduction of approximately 1 mmol/L (≈38.7 mg/dL) in LDL-C was associated with a 19% reduction in major cardiovascular events (29). In this context, considering that a 1 standard deviation increase in PHDI was associated with a − 2.8 mg/dL reduction in both TC and LDL-C in our sample, a lower cardiometabolic risk is expected over time with higher adherence.

## 5 Conclusion

We conclude that the greater adherence to PHD was associated with better lipid profile, including lower serum concentrations of TC and LDL-c and a lower number of dyslipidemia markers in children. These findings reinforce the role of sustainability-based dietary guidelines in promoting cardiovascular health from childhood. Longitudinal studies are essential to clarify causal relationships between higher adherence to PHD and the prevention of dyslipidemia in pediatric populations.

## Data Availability

The datasets are not publicly available due to confidentiality and controlled access policies. Anonymized data may be obtained from the corresponding author upon reasonable request.

## Supplementary material

SUPPLEMENTARY FIGURE 1 Prevalence of altered lipid markers in children (Viçosa, Minas Gerais, Brazil, 2015–2016). TC, total cholesterol; LDL-c, low-density lipoprotein; HDL-c, high-density lipoprotein; TG, triglycerides.

## References

[1] Faludi AA Izar MC d O Saraiva JFK Chacra APM Bianco HT Neto AA . Atualização da Diretriz Brasileira de Dislipidemias e Prevenção da Aterosclerose – 2017. Arq Bras Cardiol. (2017) 109:1–76. doi: 10.5935/abc.20170121 28813069

[2] Berenson GS Srinivasan SR Bao W Newman WP Tracy RE Wattigney WA. Association between multiple cardiovascular risk factors and atherosclerosis in children and young adults. N Engl J Med. (1998) 338:1650–6. doi: 10.1056/nejm199806043382302, 9614255

[3] Raitakari O Pahkala K Magnussen CG. Prevention of atherosclerosis from childhood. Nat Rev Cardiol. (2022) 19:543–54. doi: 10.1038/s41569-021-00647-9, 34987194

[4] Suhett LG Vieira Ribeiro SA Hermsdorff HHM Silva MA Shivappa N Hébert JR . Dietary inflammatory index scores are associated with atherogenic risk in Brazilian schoolchildren. Public Health Nutr. (2021) 24:6191–200. doi: 10.1017/S1368980021001816, 33902777 PMC11148590

[5] Faria Neto JR Bento VFR Baena CP Olandoski M Gonçalves LG d O Abreu G d A . ERICA: prevalence of dyslipidemia in Brazilian adolescents. Rev Saude Publica. (2016) 50:10s. doi: 10.1590/S01518-8787.2016050006723 PMC4767041 26910544

[6] Trautwein EA McKay S. The role of specific components of a plant-based diet in management of dyslipidemia and the impact on cardiovascular risk. Nutrients. (2020) 12:2671. doi: 10.3390/nu12092671, 32883047 PMC7551487

[7] Willett W Rockström J Loken B Springmann M Lang T Vermeulen S . Food in the anthropocene: the EAT–lancet commission on healthy diets from sustainable food systems. Lancet. (2019) 393:447–92. doi: 10.1016/s0140-6736(18)31788-4, 30660336

[8] Swinburn BA Kraak VI Allender S Atkins VJ Baker PI Bogard JR . The Global Syndemic of obesity, undernutrition, and climate change: the lancet commission report. Lancet. (2019) 393:791–846. doi: 10.1016/S0140-6736(18)32822-8, 30700377

[9] Cacau LT Benseñor IM Goulart AC Cardoso L d O Santos I d S Lotufo PA . Adherence to the EAT-lancet sustainable reference diet and cardiometabolic risk profile: cross-sectional results from the ELSA-Brasil cohort study. Eur J Nutr. (2023) 62:807–17. doi: 10.1007/s00394-022-03032-5 36266476

[10] Cacau LT Hanley-Cook GT Vandevijvere S Leclercq C De Henauw S Santaliestra-Pasias A . Association between adherence to the EAT-lancet sustainable reference diet and cardiovascular health among European adolescents: the HELENA study. Eur J Clin Nutr. (2024) 78:38093098:202–8. doi: 10.1038/s41430-023-01379-4 38093098

[11] Stubbendorff A Sonestedt E Ramne S Drake I Hallström E Ericson U. Development of an EAT-lancet index and its relation to mortality in a Swedish population. Am J Clin Nutr. (2022) 115:705–16. doi: 10.1093/ajcn/nqab369, 34791011 PMC8895215

[12] World Health Organization (WHO). Growth reference data for 5–19 years. Geneva: World Health Organization. (2007). Available online at: https://www.who.int/tools/growth-reference-data-for-5to19-years (Accessed October 23, 2023).

[13] Zabotto CB Viana R Gil MF. Registro fotográfico para Inquéritos Dietéticos: utensílios e porções. Campinas: Unicamp (1996).

[14] Tabela Brasileira de Composição de Alimentos (TBCA). Universidade de São Paulo (USP). Food Research Center (FoRC). Versão 7.0. São Paulo: Universidade de São Paulo / Food Research Center (FoRC). (2023). Available online at: http://www.fcf.usp.br/tbca (Accessed October 23, 2023).

[15] Microsoft. (2024) Sobre o power query no excel: Microsoft. Available online at: https://support.microsoft.com/pt-pt/office/sobre-o-power-query-no-excel-7104fbee-9e62-4cb9-a02e-5bfb1a6c536a (Accessed April 10, 2024)

[16] Microsoft. (2023) Introdução ao VBA no Office. Available online at: https://learn.microsoft.com/pt-br/office/vba/library-reference/concepts/getting-started-with-vba-in-office (Accessed April 23, 2023)

[17] Cacau LT De Carli E de Carvalho AM Lotufo PA Moreno LA Bensenor IM . Development and validation of an index based on EAT-lancet recommendations: the planetary health diet index. Nutrients. (2021) 13:1698. doi: 10.3390/nu13051698 34067774 PMC8156093

[18] de S FM Vieira SA Ribeiro AQ de NJF. O histórico familiar está associado à presença de dislipidemia em crianças pré-escolares. Rev Paul Pediatr. (2018) 37:41–8. doi: 10.1590/1984-0462/;2019;37;1;00005 30066825 PMC6362368

[19] Frank SM Jaacks LM Avery CL Adair LS Meyer K Rose D . Dietary quality and cardiometabolic indicators in the USA: a comparison of the planetary health diet index, healthy eating index-2015, and dietary approaches to stop hypertension. PLoS One. (2024) 19:e0296069. doi: 10.1371/journal.pone.0296069, 38198440 PMC10781024

[20] Maia JA Pinto FJ Silva FR Dantas DS Sampaio RM Chaves EM . Prevalence of dyslipidemia in children from 2 to 9 years old. Rev Bras Enferm. (2020) 73:e20190759. doi: 10.1590/0034-7167-2019-0759, 33206851

[21] Cota BC de S FM Pereira PF Juvanhol LL Novaes JF. Higher consumption of ultra-processed foods and a pro-inflammatory diet are associated with the normal-weight obesity phenotype in Brazilian children. Nutrition. (2023) 117:112234. doi: 10.1016/j.nut.2023.112234 39492097

[22] Cacau LT Benseñor IM Goulart AC Cardoso LO Lotufo PA Moreno LA . Adherence to the planetary health diet index and obesity indicators in the Brazilian longitudinal study of adult health (ELSA-Brasil). Nutrients. (2021) 13:3691. doi: 10.3390/nu13113691 34835947 PMC8625681

[23] Clemente-Suárez VJ Beltrán-Velasco AI Redondo-Flórez L Martín-Rodríguez A Tornero-Aguilera JF. Global impacts of Western diet and its effects on metabolism and health: a narrative review. Nutrients. (2023) 15:2749. doi: 10.3390/nu15122749, 37375654 PMC10302286

[24] López-Gil JF García-Hermoso A Martínez-González MÁ Rodríguez-Artalejo F. Mediterranean diet and cardiometabolic biomarkers in children and adolescents: a systematic review and Meta-analysis. JAMA Netw Open. (2024) 7:e2421976. doi: 10.1001/jamanetworkopen.2024.21976 38995643 PMC11245727

[25] Cacau LT Hanley-Cook GT Huybrechts I De Henauw S Kersting M Gonzalez-Gross M . Relative validity of the planetary health diet index by comparison with usual nutrient intakes, plasma food consumption biomarkers, and adherence to the Mediterranean diet among European adolescents: the HELENA study. Eur J Nutr. (2023) 62:2527–39. doi: 10.1007/s00394-023-03171-3, 37171585

[26] Rosi A Paolella G Biasini B Scazzina F. SINU working group on nutritional surveillance in adolescents. Dietary habits of adolescents living in North America, Europe or Oceania: a review on fruit, vegetable and legume consumption, sodium intake, and adherence to the Mediterranean diet. Nutr Metab Cardiovasc Dis. (2019) 29:544–60. doi: 10.1016/j.numecd.2019.03.003, 31078365

[27] Miller V Mente A Dehghan M Rangarajan S Zhang X Swaminathan S . Fruit, vegetable, and legume intake, and cardiovascular disease and deaths in 18 countries (PURE): a prospective cohort study. Lancet. (2017) 390:2037–49. doi: 10.1016/S0140-6736(17)32253-5, 28864331

[28] Cacau LT Souza TN da Costa Louzada ML Marchioni DM. Adherence to the EAT-lancet sustainable diet and ultra-processed food consumption: findings from a nationwide population-based study in Brazil. Public Health Nutr. (2024) 27:e183. doi: 10.1017/S1368980024001678 39363443 PMC11505050

[29] Wang N Fulcher J Abeysuriya N Park L Kumari A Di Tanna GL . Intensive LDL cholesterol-lowering treatment beyond current recommendations for the prevention of major vascular events: a systematic review and meta-analysis of randomised trials including 327037 participants. Lancet Diabetes Endocrinol. (2020) 8:36–49. doi: 10.1016/S2213-8587(19)30388-2, 31862150

