# Supplementary figures and images for: Greater adherence to the Planetary Health Diet is inversely associated with dyslipidemia in children: PASE Study—Brazil
Source: Front Nutr. 2026 Mar 12;13:1684547. doi: 10.3389/fnut.2026.1684547 (PMC13017257; doi:10.3389/fnut.2026.1684547)

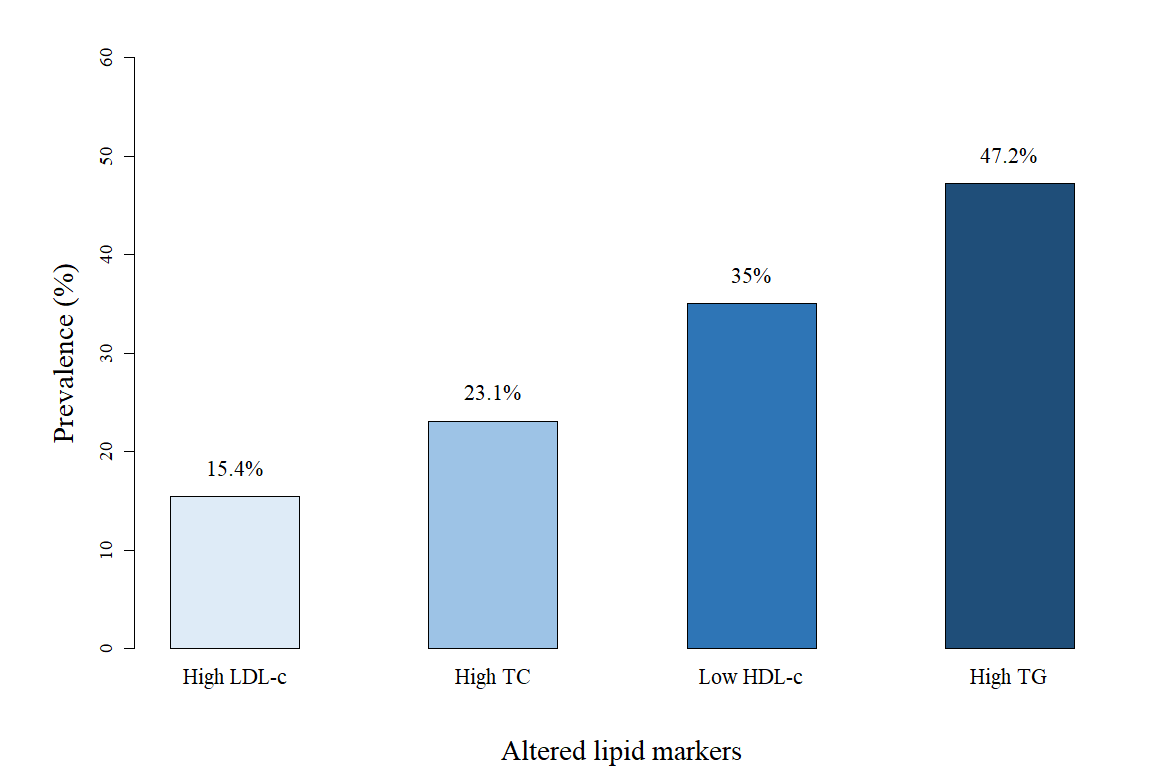

Supplement: SUPPLEMENTARY FIGURE 1 — Prevalence of altered lipid markers in children (Viçosa, Minas Gerais, Brazil, 2015–2016). TC, total cholesterol; LDL-c, low-density lipoprotein; HDL-c, high-density lipoprotein; TG, triglycerides. [file Image_1.tiff]
